# Laboratory-based measures of executive function and daily living skills in young children with Down syndrome: informing future interventions

**DOI:** 10.1111/jir.13176

**Published:** 2024-08-07

**Authors:** K. Van Deusen, M. A. Prince, M. M. Walsh, A. J. Esbensen, L. R. Patel, M. E. Pinks, A. J. Thurman, L. Abbeduto, L. A. Daunhauer, D. J. Fidler

**Affiliations:** 1 Department of Human Development and Family Studies, Colorado State University, Fort Collins, CO, USA; 2 Division of Developmental and Behavioral Pediatrics, Cincinnati Children’s Hospital Medical Center, Cincinnati, OH, USA; 3 College of Medicine, University of Cincinnati, Cincinnati, OH, USA; 4 Department of Psychiatry, University of Colorado Anschutz Medical Campus, Aurora, CO, USA; 5 MIND Institute, University of California Davis, Sacramento, CA, USA; 6 Department of Psychiatry and Behavioral Sciences, University of California Davis Health, Sacramento, CA, USA

**Keywords:** adaptive behaviour, clinical trials, Down syndrome, executive function, secondary endpoints

## Abstract

**Background:**

Adaptive behaviour refers to the practical skills necessary for independence and is considered a high-priority intervention target for children with neurogenetic conditions associated with intellectual disability, like Down syndrome (DS). Daily living skills (DLS) are a critical aspect of adaptive behaviour, but they have received little intervention attention, possibly because they involve a wide variety of skills across many settings. The present study aimed to advance DLS intervention science by examining the concurrent and longitudinal association between DLS performances and a cognitive skillset hypothesised to support DLS skill acquisition, executive function (EF).

**Methods:**

Participants were 71 children with DS between the ages of 2.5 and 8.7 years (M = 5.23 years; standard deviation = 1.65) who completed a battery of adapted EF tasks and a primary caregiver who completed the Vineland Adaptive Behavior Scales 3rd Edition Parent/Caregiver Comprehensive Report Form. A subset of caregivers also provided 6- and 12-month follow-up adaptive behaviour information.

**Results:**

Results demonstrated a positive association between EF task performance and DLS standard scores and *v*-scores both concurrently and longitudinally.

**Conclusions:**

The findings have implications for potential future intervention approaches that aim to strengthen DLS performances by advancing EF skills in this population.

## Introduction

Individuals with Down syndrome (DS) are predisposed to challenges with the practical daily activities necessary for personal independence, or ‘adaptive behaviour’ (Daunhauer 2011). Adaptive behaviour is a high-priority target for treatment and intervention in DS (Esbensen *et al*. 2017; Baumer *et al*. 2022), and within this domain, gaining proficiency with ‘daily living skills’ (DLS) is particularly important for independence throughout development. Although some investigations identify DLS as an area of relative strength when compared with other aspects of adaptive behaviour in DS (Dykens & Hodapp 1994) and others have found DLS an area of relative challenge, all studies report delays in this domain relative to chronological age (CA)-matched peers (see Daunhauer 2011 for synthesis). Recent studies of DLS in children and adolescents with DS report average scores more than two standard deviations (SDs) below the mean (Spiridigliozzi *et al*. 2019; Will *et al*. 2021; Onnivello *et al*. 2022a; Schworer *et al*. 2022a), representing a notable delay in these functional skills relative to peers without DS.

Although existing intervention paradigms have targeted adaptive social and communication skills in children with neurodevelopmental conditions (Yoder & Stone 2006; McDuffie *et al*. 2012; Burns *et al*. 2019; Fuller *et al*. 2020; Waddington *et al*. 2021), remarkably few have focused on DLS (Bishop & Pangelinan 2018). A review published in 2019 that synthesised 50 years of research on DLS interventions for people with intellectual disability (ID) identified only 20 studies for review (Burns *et al*. 2019), and of the 20 studies identified, only three included children under the age of 8 years, one of which was a single-case intervention study (Burns *et al*. 2019). Another comprehensive review of 20 years of behaviour analysis interventions for children with ID identified 49 peer-reviewed studies with a range of communication and social targets (word fluency, vocal responses and imitation), but only one study listed adaptive behaviour as the primary outcome of interest (Ho *et al*. 2021). The relative paucity of DLS intervention innovation for children with ID is confounding, especially given the importance of these skills for functional outcomes. One explanation may be that addressing DLS via intervention is particularly challenging because it encompasses many different activities, settings and temporal contexts. Developing comprehensive interventions that promote a vast array of DLS may be prohibitive in terms of cost and feasibility. Yet this area remains a high-priority intervention target in DS and other conditions associated with ID, and innovations to support the development of these skills are needed.

Identifying the developmental constructs that underlie DLS acquisition and strengthening these skills is a promising alternative approach to promoting outcomes in this area. Such an approach would involve targeting underlying skills that facilitate learning across a range of contexts and interactions in naturalistic environments. One potential candidate for such interventions is ‘executive function’ (EF), or the cognitive regulatory processes necessary for goal-directed behaviour. EF includes several component processes, including working memory, inhibition and cognitive flexibility, that are thought to work in concert to promote planning and organisation across a wide variety of contexts (Miyake *et al*. 2000; Diamond 2013). *Working memory* refers to the temporary storage and manipulation of information during tasks or goal pursuit; *inhibition* refers to the ability to resist prepotent thoughts and behaviours in favour of more considered responses; and *flexibility* refers to the ability to adapt thoughts and behaviours based on incoming fluctuations in the environment. These three components of EF have been shown to predict important outcomes in the general population, including adaptive behaviour, academic achievement, health and employment (Best & Miller 2010; Moffitt *et al*. 2011; Harms *et al*. 2014), and they are predictive of many aspects of adaptation during early childhood (Diamond 2006, 2013; Garon *et al*. 2008).

### Executive function in Down syndrome

DS is associated with EF challenges throughout all phases of the lifespan (Tungate & Conners 2021), and challenges in EF are generally more pronounced than would be expected based on overall developmental status (Lee *et al*. 2011; Daunhauer *et al*. 2014). Individuals with DS demonstrate different patterns of strength and challenge with the subcomponent processes of EF across the lifespan (Lee *et al*. 2011; Daunhauer *et al*. 2014; Loveall *et al*. 2017; Onnivello *et al*. 2022b), and within-group heterogeneity is observed along this dimension as well (Van Deusen *et al*. 2022). Working memory is consistently reported as an area of greater challenge, and inhibitory control and cognitive flexibility show greater variation by developmental stage (Daunhauer *et al*. 2014; Loveall *et al*. 2017). A recent mixture modelling analysis of children with DS ages 3–10 years old identified two profiles of EF challenges: one profile with clinically significant challenges in working memory only, and another with clinically significant challenges across several EF domains (Van Deusen *et al*. 2022). EF challenges impact a range of outcomes in DS, including academic achievement, employment and functional performance (Will *et al*. 2017; Tomaszewski *et al*. 2018). Given the critical role of EFs in driving goal-directed behaviour across a range of domains, these cognitive skills are potentially important candidates for future DLS intervention innovation in DS. To date, however, a longitudinal evidence base for the relation between childhood EF and DLS outcomes that could justify such an approach has not yet been reported in DS research.

### Adaptive behaviour and executive function in Down syndrome

Examining the association between adaptive behaviour and EF in children with DS requires several considerations. First, although adaptive behaviour is almost always measured via proxy report, EF can be measured through either direct observation or proxy report, with each approach capturing different aspects of EF (Toplak *et al*. 2013). Laboratory-based performances are thought to represent an individual’s raw efficiency of processing, while proxy-report measures likely capture aspects of everyday goal pursuit (Toplak *et al*. 2013). From an intervention design perspective, improvement in the raw efficiency of processing is a potential mechanism that would lead to more effective goal pursuit and therefore facilitate mastery of activities of daily living. A link between EF and adaptive behaviour has been reported in recent mixture modelling work in a cohort of children and adults with DS (Channell *et al*. 2021) and through a linear cross-sectional study design involving children (Onnivello *et al*. 2022a). However, to date, the evidence base has relied on the use of a proxy report of EF to demonstrate an association with DLS in school-age children with DS, and this association was reported only in children older than 6 years.

### Current study

The present study examined the association between laboratory-based EF performances in children with DS and caregiver-reported DLS concurrently and longitudinally. Children with DS participated in working memory, inhibition and cognitive flexibility tasks that were adapted to include minimal demands on expressive language, receptive language and motor planning, all areas vulnerable to significant delay in DS (Esbensen *et al*. 2022). These adaptations thereby reduce interpretational confounds in this population. Caregivers completed the Vineland Adaptive Behavior Scales 3rd Edition Parent/Caregiver Comprehensive Report Form (VABS-3) concurrently with the EF assessment, and a subset of caregivers also completed the VABS-3 at follow-up visits 6 and 12 months later. The associations between EF laboratory performances and both concurrent and later DLS scores were evaluated.

## Methods

### Participants

Participants were 71 children with DS between the ages of 2.5 and 8.7 years (Mean (M) = 5.23 years; Standard deviation (SD) = 1.65) and a primary caregiver. Children were 50.7% male (*n* = 36). The majority of participants were White and non-Hispanic or Latino. Table 1 includes a description of the participant demographics. A subgroup of 47 caregivers participated in a second data wave, which was called ‘visit 2’, 6 months after the initial data wave, labelled as ‘visit 1’. Additionally, 39 caregivers took part in a third data wave (‘visit 3’), which was conducted 12 months after visit 1.

Overall, few differences were observed between those who did and did not return for follow-up visits. No significant differences were observed between participants who completed only visit 1 compared and those who completed both visits 1 and 2 on the dimensions of CA, mental age (MA), race, ethnicity or DS type (*t*s = −0.61 to 1.80; *P*s = 0.086–0.543). Caregivers of females were more likely to return for a 6-month follow-up visit (*χ*^2^(1, *N* = 71) = 5.88; *P* = 0.015). There were no significant differences for the dimensions of CA, MA, sex, race, ethnicity or DS type between participants with caregivers who completed visit 1 only compared with those who completed visits 1 and 3 (*t*s = 0.15–1.39; *P*s = 0.171–0.880; *χ*^2^(1, *N* = 71) = 3.24; *P* = 0.072).

### Procedures

Participants were recruited into a multi-site study on EF in children with DS from three regions in the USA. All participating caregivers and children completed the consent process prior to beginning project procedures. All procedures were approved by a multi-site institutional review board. Inclusion criteria for participation included English understanding for the caregiver and participant, corrected hearing and vision problems, and 1 month of stable medication use (if children were prescribed medications). English was not required as a child’s primary language; however, only those caregivers who reported comfort answering questions in English were included in the present study.

Participants completed an initial visit with the study team that involved both child assessment and caregiver questionnaires (visit 1). Research visits were held in laboratory spaces available to the multi-site research team, and caregivers were asked to complete questionnaires during direct assessment activities. A subset of participants returned for visits 2 and 3. For most participant visits (91.5%, *n* = 65), COVID-19-related safety precautions were in place, including the use of face masks, face shields and/or eye shields. Institutional and municipal guidelines for the responsible conduct of research were implemented during the COVID-19 pandemic. Study data were collected and managed using REDCAP electronic data software hosted at the Colorado Clinical and Translational Sciences Institute (Harris *et al*. 2009, 2019).

All measures were assessed by trained graduate or professional research associates. Examiners were evaluated annually for adherence to fidelity of administration, with a requirement of reaching 90% fidelity. Ongoing support was provided across sites to ensure consistency in administration.

### Measures

#### Caregiver report measures

##### Demographics questionnaire.

At the initial visit, caregivers completed a questionnaire pertaining to their child’s sex as assigned at birth, race and ethnicity. Caregivers also reported on their own age, education attainment and household income.

##### Biomedical history questionnaire.

At visit 1, caregivers provided information regarding their child’s medical history. Questions were asked about the presence of co-occurring conditions and diagnoses including prematurity, congenital heart defects, biomedical conditions (e.g. thyroid problems and gastrointestinal concerns), sensory challenges (e.g. vision and hearing problems) and psychiatric diagnoses (e.g. attention-deficit hyperactivity disorder and autism spectrum disorder).

##### Vineland Adaptive Behavior Scales 3rd Edition Parent/Caregiver Comprehensive Report Form (VABS-3; Sparrow *et al*. 2016).

The VABS-3 is a caregiver-completed measure of adaptive behaviour for individuals ages birth to 99 years. This tool has been widely used to assess socialisation, communication, daily living and motor skills. Each item is answered with a Likert-type scale for respondents to answer if the participant ‘usually’, ‘sometimes’ or ‘never’ performs a behaviour independently. Domain standard scores (SSs) are generated from raw scores that have a mean of 100 and SD of 15. Subdomains of each domain score have *v*-scale scores, which are standardised from raw scores. The *v*-scale scores have a mean of 15 and SD of 3. SSs and *v*-scale scores were used in this investigation to examine associations with EF task performances.

The VABS-3 has strong established reliability (*r*s = 0.91–0.99) across all domains of the parent/caregiver comprehensive form. The VABS-3 has also demonstrated validity with moderate to strong correlations to the Bayley-3 Adaptive Behavior (*r*s = 0.60–0.81; Sparrow *et al*. 2016) and the Adaptive Behavior Assessment System-3 (*r*s = 0.46–0.76; Sparrow *et al*. 2016). The Daily Living Skills Scale has demonstrated strong validity with each of these tools [Bayley-3 Adaptive Behavior (*r* = 0.67) and Adaptive Behavior Assessment System-3 General Adaptive Composite (*r* = 0.46)].

The DLS domain measures performance on practical, everyday tasks of living adjusted for what would be expected for the participant’s CA. The present study focused on participants’ reported DLS, which include personal (e.g. eating, hygiene and dressing), domestic (e.g. cleaning up after themselves)/numeric (e.g. practical uses of numeric concepts, including time and money) and community skills (e.g. safety and responsibility outside of the home)/school community (e.g. appropriate behaviour in school environment) subdomains (Sparrow *et al*. 2016). The *v*-scale scores cannot be calculated for the subdomains of domestic and community for participants younger than 3-year CA.

At all three visits, caregivers completed the VABS-3 most often in Q-global, an online assessment questionnaire platform developed and administered by the test publisher and, in rare instances, on paper report forms. Project-standardised assessment procedures included starting at the 1-year start point. Associations with the Adaptive Behaviour Composite (ABC) SS are reported; however, only the DLS domain of adaptive behaviour was the focus of this investigation.

#### Child assessment developmental status measures

##### Bayley Infant and Toddler Scales of Development – 4th Edition (Bayley & Alyward 2019).

The Bayley Infant and Toddler Scales of Development – 4th Edition (Bayley-4) is a measure of developmental status for children aged 1–42 months. The Bayley-4 examines three subdomains: cognition, cand motor skills. The Bayley-4 is a validated standardised assessment measure and demonstrates strong internal consistency in DS (*r* = 0.98; Bayley & Alyward 2019). This measure has been used to assess children with DS (Pinks *et al*. 2023; Van Deusen *et al*. 2023; Walsh *et al*. 2023).

##### Stanford–Binet 5th Edition: Abbreviated Intelligence Quotient (Roid 2003a).

The Stanford–Binet 5th Edition: Abbreviated Intelligence Quotient (SB5-ABIQ) is used for individuals ages 2–85 years to measure IQ. The SB5-ABIQ is comprised of performance from two subtests: verbal knowledge and nonverbal object series/matrices. Raw scores were transformed into MA estimates and a standardised IQ estimate. The SB5-ABIQ has strong internal consistency with the other subtests in the measure (above 0.90; Roid 2003b) and is increasingly used in DS research (Schworer *et al*. 2021; Schworer *et al*. 2022b; Pinks *et al*. 2023; Soltani *et al*. 2023; Van Deusen *et al*. 2023; Walsh *et al*. 2023).

MA estimates were calculated for each participant based on the administration of either the SB5-ABIQ (Roid 2003a) or the Bayley-4 (Bayley & Alyward 2019). Children older than 3 years were administered the SB5-ABIQ, and children 2.5–2.99 years were only assessed via the Bayley-4. A subset of children, 3–4.99 years, completed the Bayley-4 in addition to the SB5-ABIQ, time-permitting. This procedure to extend variability by using ‘out of age level testing’ has precedence in work with children with ID (Thurm *et al*. 2020) and DS (Pinks *et al*. 2023; Van Deusen *et al*. 2023; Walsh *et al*. 2023). In the present study, the Bayley-4 increased the sample variability in MA estimates when children scored on the floor of the SB5-ABIQ. When possible (*n* = 22), the MA from the Bayley-4 was used in place of the floor MA score on the SB5-ABIQ, which is <24 months. There were six instances in which an MA estimate was not obtained. The reasons for missing these observations included participant refusal or fatigue and the time limitations of the assessment.

#### Child assessment executive function measures

##### Working memory: Garage Game task (Pinks *et al*. 2023).

The Garage Game is an adapted self-ordered pointing task designed to evaluate working memory updating in young children. Child participants were presented with a set of toy cars and colour-corresponding toy garages. They were asked to find the location of each car hidden in the garages. Correct responses involved remembering the cars that were found in previous trials and searching in new locations to find the remaining cars. Instructions to the child were ‘Find a car!’ or ‘Where’s a car?’, which reduced receptive language requirements and conveyed the task goal of finding hidden cars. When an empty garage was selected, participants received feedback to try again (e.g. ‘This garage is empty. Let’s try again!’). The task was discontinued if children selected three empty garages in a row.

At the start of the task, children watched or helped the examiner ‘park’ the cars in each garage, followed by the examiner closing the garage doors simultaneously. During practice, children identified the location of the three cars in the garage, with the found cars visible on the table. In the test trials, children were presented with the same garage as the practice trial, but a distractor screen was introduced ahead of the participant selecting the next garage. Additionally, upon finding a car in test trials, the car was hidden from sight of the participant before they made their next choice. If a child selected an empty garage during the test trials, examiners said, ‘Oh, it’s not there. Let’s try again’. Children continued to select cars until they found each of the three hidden cars or selected three empty garages consecutively. A second test trial with the same three-car garage was completed. A third and final set included the familiar three-car garage and a new three-car garage, providing six cars for the children to find.

The feasibility and developmental sensitivity of this measure have been demonstrated for children with DS ages 2.5–8 years (Pinks *et al*. 2023, Van Deusen et al., under review). Preliminary test–retest reliability was moderate [intraclass correlation coefficient (ICC) = 0.60] for the repetitive search rate (Pinks *et al*. 2023). Per Pinks *et al*. (2023), the repetitive search rate was defined as the quotient of the total number of incorrect (empty garage) searches by the participant divided by the number of cars the participant had the opportunity to find. Lower repetitive search rates indicate more advanced working memory skills. Scoring was conducted *in vivo* by the examiners. Two participants were missing scores for this assessment due to refusal.

##### Inhibitory control: Snack Delay task (Kochanska *et al*. 1996, 2000).

The Snack Delay task is a measure of inhibitory control designed to measure early response inhibition in 21- to 42-month-old children without developmental delays. In the original paradigm, children were asked to wait to retrieve a desirable snack under a transparent cup until the examiner rang the bell at the end of each trial (10, 20, 30 and 15 s, respectively; Kochanska *et al*. 1996, 2000). Children were scored based on eating the snack immediately, eating it after the examiner lifted the bell or waiting for the examiner to ring the bell. Adaptations to the original task for administration with children with DS involved shortening the wait times (5, 10, 20 and 15 s); the examiner did not lift the bell during the waiting period of administration; and examiners were permitted to share nonverbal engagement (eye gaze and facial displays) with the child participants during each trial. Children were presented with a snack or toy and then asked to wait to retrieve the toy until the examiner said, ‘Go!’ and rang the bell. The snack or toy was hidden under a clear cup and the child was able to view it throughout the task. Children were told, ‘Don’t eat the snack. Remember, wait until I ring the bell.’

*In vivo* scoring included the examiner’s appraisal of the children’s motivation or enthusiasm for the toy. This was evaluated based on the participant’s interest in moving towards the snack/toy, wanting more snack/toy play, and social cues of sharing, smiling and requesting. Coding for this task used the observer xt Noldus computer software to time children’s waiting and observe the frequency of behaviours outside of the task goal (Noldus Information Technology 2013). Two coders naïve to the study objectives were trained to reliability in scoring this assessment (inter-rater reliability = 0.98). The mean latency to producing any dysregulated behaviour (e.g. poking at or tapping the cup, picking up the cup, playing with the bell and engaging in other off-task behaviour) and frequency of dysregulated behaviour were calculated. Mean latency to dysregulated behaviour was the primary variable included in analyses. The task was feasible to administer with 97.2% of participants engaging with the task. The snack delay task has demonstrated construct validity with other measures of inhibitory control that have been recently evaluated (Van Deusen et al. under review).

##### Cognitive flexibility: Adapted Reverse Categorisation task (Carlson *et al*. 2004; Van Deusen *et al*. 2023).

Participants completed a reverse categorisation task adapted from Carlson *et al*. (2004) wherein children sorted objects into two locations based on size. In the adapted task, children sorted two toys that were different shapes and high-contrast colours to support different levels of visual acuity. In the first trial, children sorted by a colour-congruent rule (e.g. ‘These red blocks are ‘ketchup’ and these yellow balls are ‘mustard’. I want you to put the ketchup in the ketchup bucket *[red bucket]* and the mustard in the mustard bucket *[yellow bucket]*’). When participants sorted six or more items correctly in the colour-congruent trial, they moved on to the ‘silly game’ to sort colour-incongruent. The instructions for colour-incongruent items were as follows: ‘Now we are going to play the silly game! I want you to put the mustard in the ketchup bucket and the ketchup in the mustard bucket. Let’s try one!’ Children sorted up to 10 items with each rule, but the task was discontinued if they incorrectly sorted three items in a row at any point during participation. This measure has demonstrated greater than 90% feasibility for young children with DS and the greatest sensitivity for children who have MAs between 1 and 3 years and CAs 4–7 years (Van Deusen *et al*. 2023). The correct number of sorts (up to 10) following the rule change was scored *in vivo* by the examiner. A preliminary evaluation of test–retest reliability for this measure indicated reliability (ICC = 0.81) in children with DS (Van Deusen *et al*. 2023).

### Analytic approach

The analytic plan for this paper involved predicting domain SSs and subdomain *v*-scores of the VABS-3 DLS from performances on three adapted EF tasks. First, descriptive analyses (mean, SD, minimum and maximum) were calculated for all EF task performance variables and the DLS scores of interest (SSs and *v*-scores). Spearman’s rho correlations were calculated to examine the associations between key indicators of EF performance and the subdomain *v*-scores of DLS (i.e. personal, domestic and community). Nonparametric correlations were calculated to address the non-normality in the distributions of EF measures. Correlation matrices were generated for each time point (i.e. visit 1, visit 2 and visit 3). Missing data points were removed with a list-wise deletion from analyses. In three separate regression equations, the SSs of DLS were regressed on participant performance on each of the EF tasks (i.e. working memory, inhibitory control and cognitive flexibility). One regression model was generated for each time point (visit 1, visit 2 and visit 3) to examine EF tasks as predictors of DLS scores at different lengths of time from the EF assessment. CA was used as a preliminary covariate in the regression models; however, there were no discernible effects of age, and it was removed from the reported analyses.

## Results

### Descriptive analyses

#### Executive function task performance

The EF tasks demonstrated high feasibility and a wide range of performances across child participants (Table 2). There were 62 participants (87.3%) who passed the Garage Game pre-test. The average repetitive search rate for participants in the sample was 0.39 (SD = 0.39; range: 0.00–1.67). Of the 71 participants who completed the inhibition Snack Delay task, 59 (83.1%) demonstrated motivation towards reaching for the snack or toy. On average, the latency to produce a dysregulated behaviour was 6.73 s (SD = 5.88; range: 0.03–20.51 s). The flexibility task (Adapted Reverse Categorisation) was attempted by the entire sample, with 51 children successfully sorting six or more toys in trial 1. The average number of correct sorts on the colour-incongruent trial was 4 toys out of 10 (SD = 4.56; range: 0–10). Working memory repetitive search rate was negatively correlated with cognitive flexibility post-switch score (*r*(62) = −0.50, *P* < 0.001). Working memory was also significantly negatively correlated with inhibition (*r*(62) = −0.28, *P* = 0.031). No other associations were observed among the EF tasks.

#### Adaptive behaviour

Participants demonstrated a wide range of scores on the ABC SS, the DLS SS and the *v*-scores of the DLS. The average VABS-3 ABC SS was 72.43 (SD = 6.85; range: 52–88) at visit 1, with an average DLS SS of 70.65 (SD = 9.63; range: 50–92). Scores were similar at visits 2 and 3, with a slight decline in average SSs over time. A full distribution of performances on the ABC SS, DLS SS and DLS *v*-scores is presented in Table 3.

### Associations between daily living skills subdomains and executive function task performances

Spearman rank-order correlations were calculated to examine the associations between EF task performance and the subdomains of the DLS domain of adaptive behaviour. A complete matrix for all three time points can be found in Table 4. Degrees of freedom varied based on attrition at follow-up assessments, and two of the three EF tasks required passing a pre-test to move on to test trials.

Performances on the three EF tasks were significantly associated with several DLS subdomain *v*-scores. Inhibition performances (mean latency to dysregulated behaviour) were significantly associated with each DLS subdomain at every time point (*r*s = 0.29–0.53, *P* < 0.05). Working memory performances were significantly negatively associated with the personal subdomain of DLS at all three visits (*r*s = −0.25 to −0.46, *P* < 0.05) and negatively related to the domestic and community subdomains of DLS at visit 3 (*r*s = −0.38 and −0.52, *P* < 0.05). These negative associations were in the expected direction (lower scores on the working memory task indicate less repetitive searching behaviour and stronger working memory skills). Cognitive flexibility performances were associated with DLS *v*-scores at visits 1 and 3, but not at visit 2. Personal (*r*(69) = 0.26; *P* = 0.033) and domestic (*r*(62) = 0.25; *P* = 0.050) *v*-scores were associated with cognitive flexibility performances at visit 1. All three EF task performances were more strongly associated with DLS subdomain *v*-scores at visit 3 (*r*s = |0.33|–| 0.53|, *P* < 0.05) than visit 1. Working memory and cognitive flexibility were also more strongly associated with DLS SS at visit 3 than visit 2, though inhibitory control was comparably associated with DLS SS at visits 2 and 3.

### Executive function task performance and daily living skills standard scores

Multiple regression analyses were used to examine the relationship between performances on the three EF laboratory measures and caregiver-reported DLS SS concurrently and longitudinally (Table 5). The concurrent regression model, *F*_3, 57_ = 5.02, *P* = 0.004, *R*^2^ = 0.21, explained 21% of the variance in DLS performances. Performances on the inhibitory control task demonstrated the strongest relation to DLS SS (*β* = 0.40; *P* = 0.003), and cognitive flexibility was a significant predictor as well (*β* = 0.28; *P* = 0.047). Working memory task performance was not predictive of concurrent DLS SS (*β* = 0.15; *P* = 0.326). In the regression model that predicted DLS SS at visit 2, *F*_3, 36_ = 6.63, *P* = 0.001, *R*^2^ = 0.36, EF performances explained a greater amount of variance (36%) than in the concurrent model, with inhibitory control as a significant predictor of DLS SS (*β* = 0.56, *P* < 0.001). In the estimation model for DLS SS from EF assessment 12 months prior, *F*_3, 31_ = 6.00, *P* = 0.002, *R*^2^ = 0.37, an even greater amount of variance was explained by the EF predictors at visit 3 (37%) than from either of the first two time points. Inhibitory control performance was a significant predictor in this model (*β* = 0.33; *P* = 0.050; Table 5).

## Discussion

This study aimed to evaluate the association between laboratory-based EF measures and activities of daily living in children with DS concurrently and longitudinally. DLS was selected as a focus of investigation because of the centrality of these skills for independence and its relatively sparse intervention evidence base among people with ID. Correlation and regression analyses demonstrated significant associations between laboratory-task performances and adaptive behaviour DLS scores both concurrently and longitudinally, with EF task performance accounting for approximately 37% of the variance in DLS outcomes 1 year later. These longitudinal findings are potential evidence of a mechanistic role in DLS acquisition and can potentially shape novel intervention approaches for children with DS.

There was a notable increase in the amount of variance explained by EF task performance across 1 year for DLS. This increase from 21% concurrently to 37% 1 year following EF assessment suggests that EF skills may play a foundational role in the development of independence for DLS. It was notable that there was differential predictability between EF subcomponents and DLS in each of the regression analyses. This complexity warrants additional investigation to understand how different subcomponents of EF may uniquely contribute to opportunities to develop independence with DLS. The findings presented were independent of CA associations, strengthening the potential argument for EF skills as a foundation for DLS.

The associations presented in Table 4 highlight several important patterns between specific subcomponent processing of EF and the subdomains of DLS in children with DS. First, inhibitory control was most consistently associated with each DLS subdomain *v*-score at all three visits. Inhibitory control, measured in this study via delay of gratification, was associated with skills of personal, domestic and community adaptive behaviour. Working memory was more predictive of each DLS subdomain 1 year from EF assessment than at either concurrent or 6-month follow-up DLS reports. The stronger longitudinal association may suggest that working memory is foundational for developing more advanced DLS skills over time. For example, children with a foundation of updating skills may more easily remember a goal and update their progress towards that goal than children who are working to link together emerging skills into the completion of activities of daily living.

The final subcomponent EF skill, cognitive flexibility, was more strongly related to DLS *v*-scores at visit 3 than visit 1 or visit 2. Small significant correlations were found between cognitive flexibility and personal and domestic *v*-scores at visit 1, and stronger associations emerged at visit 3 for all three DLS subdomains. This suggests that cognitive flexibility may be similarly foundational to developing independence with DLS skills. Cognitive flexibility is an essential skill for being responsive to changing environments and staying on track to complete tasks. The pattern observed with cognitive flexibility is comparable with that of working memory, which is notable because an investigation in the same age range has found that working memory and cognitive flexibility load onto the same factor in children with DS (Van Deusen et al. under review). These patterns of associations provide evidence from early childhood that EF processing is related to DLS concurrently and over the course of 1 year.

### Intervention implications in Down syndrome

Adaptive behaviour challenges are a core feature of ID, and consequently, they are an important target for novel interventions and treatments in DS, particularly during childhood (Thurm *et al*. 2020). Although numerous intervention approaches have aimed to strengthen the adaptive use of social and communication skills, few developmentally focused interventions have targeted DLS as an outcome. Rather than developing interventions that focus on each activity of daily living individually, a potentially parsimonious approach may be to strengthen underlying cognitive regulatory processes that support the acquisition of DLS. The well-documented association between EF and adaptive behaviour in the general population and in other clinical populations suggests that EF foundations could be a promising mechanism for facilitating adaptive behaviour skill acquisition in DS (Gilotty *et al*. 2002; Harms *et al*. 2014; Duncan & Bishop 2015; Pugliese *et al*. 2016).

To date, the evidence to justify such an approach has relied only on proxy-reported EF, which has limitations related to reporter bias (Esbensen *et al*. 2021). Historically, there has been limited investigation into the utility of childhood laboratory measures of EF for use in DS (Schworer *et al*. 2022b; Schworer *et al*. 2023; Soltani *et al*. 2023). Recent work, however, has demonstrated the psychometric strength of laboratory measures of early EF that are ‘syndrome-informed,’ involving adaptations to EF laboratory tasks in ways that reduce receptive language, eliminate expressive language demands and simplify motor requirements (Daunhauer & Fidler 2011; Pinks *et al*. 2023; Van Deusen *et al*. 2023; Walsh *et al*. 2023). Preliminary findings from this contemporary evaluation work have demonstrated that, by making syndrome-informed adaptations to commonly used measures of early EF in the general population, such measures demonstrate feasibility, developmental sensitivity and preliminary evidence of test–retest reliability (Pinks *et al*. 2023; Van Deusen *et al*. 2023; Walsh *et al*. 2023). Findings from the present study demonstrated that child performances on adapted laboratory EF measures are positively associated with emerging DLS in young children with DS. Establishing an association between DLS and performances on these validated EF laboratory tasks strengthens the rationale for the development of cost-effective and feasible EF-focused interventions designed to improve DLS outcomes across a wide range of domains.

It is notable that a model consisting of three brief, game-based EF tasks was predictive of approximately one-third of the variance in DLS outcomes, with the amount of variance explained increasing over the course of 1 year. Although additional factors most assuredly contribute to the DLS outcomes, these EF findings are notable in the degree to which they are associated with real-world outcomes, as reported by caregivers who were not influenced in their responses by their child’s laboratory-based performances. Additional factors, including motor skills, other aspects of cognition, co-occurring conditions, early learning and intervention opportunities, and aspects of the home environment, all likely contribute to the remainder of the variance in skill acquisition; however, the strong association that has been reported elsewhere between EF and adaptive behaviour was once again observed in this study. These promising findings not only have implications for outcome measure selection, but they also extend the evidence base for an association between EF and adaptive behaviour to younger CAs in DS.

Further psychometric and measurement insights related to early EF measurement in DS were generated from the present study. Although ‘task impurity’ is often a challenge in EF laboratory assessments, the present measures were selected for adaptation because they featured one key EF component prominently and reduced other confounds of developing skills. The intended minimisation of construct overlap in measurement is evident in that only one moderately significant association was observed across the three measures (working memory and cognitive flexibility). The small significant correlation between working memory and inhibition suggests that the tasks may have captured some overlapping skills, but not extensively so. Task performances on the three EF measures were not otherwise significantly related. Such minimal associations between EF task performances suggest that it is worthwhile to use several laboratory measures collectively when considering EF as a target for future DLS-related interventions.

### Study limitations

Findings from the present study can be considered part of the effort to advance intervention research that focuses on practical DLS in DS (Esbensen *et al*. 2017; Thurm *et al*. 2020; Baumer *et al*. 2022). These findings, however, should be interpreted in the context of several study limitations. First, although the study sample was relatively large within DS research, analyses were limited by the number of participants who completed follow-up visits. A larger sample for the longitudinal analyses would provide greater support for the implications of EF performance as a predictor of future DLS. Future replication of the study findings with additional cohorts is warranted. It is also important to consider that the project period overlapped with the COVID-19 pandemic. Examiners took appropriate precautions to protect participants in research visits whenever possible, but recruitment may have been skewed towards families who felt comfortable with participation or did not face additional burdens or barriers to community engagement during the pandemic.

Another limitation of the present study relates to the representativeness of the participants in the study sample. For the identification of meaningful outcomes across the full population of individuals with DS and ID, future studies should implement recruitment and community-engagement procedures that will lead to greater participation by individuals from minoritised identity groups. The measurement of adaptive behaviour for this investigation was also completed via caregiver report. Although this is a common practice for assessing adaptive behaviour, including in the diagnosis of ID, there are limitations to caregiver report. Caregivers may interpret questionnaire items differently, which may limit the interpretation of skill acquisition for children with DS.

This investigation focused on the association between EF and DLS but did not include the other domains of adaptive behaviour. Future studies should further examine the association between EF performances and the other subdomains of adaptive behaviour (i.e. socialisation, communication and motor) and the role of EF in the application of practical social and communication skills in real-world settings. Such examinations may further inform adaptive behaviour intervention development in ways that facilitate independence during activities of daily living.

Although the earlier caveats should be taken into account when considering the implications of these findings, the present study advances our current understanding of the relationship between EF laboratory-task performances and caregiver report of DLS in children with DS, both concurrently and longitudinally. These findings, if replicated, can set the stage for intervention innovation that aims to improve independence in activities of daily living by targeting underlying cognitive regulatory processes necessary for goal-directed behaviour. Novel intervention approaches that consider the developmental underpinnings of DLS are a promising way forward, and may ultimately increase well-being and adaptation for people with DS throughout their lifespan.

## Figures and Tables

**Table 1:** Demographic information

Child variable	% (*n*) or M (SD)
% male (*n* = 3 missing)	50.7 (36)
Child chronological age (years)	5.23 (1.65)
Child developmental age (years)	2.45 (0.90)
Race (*n* = 5 missing)	
American Indian or Alaska Native	1.5 (1)
Asian American	1.5 (1)
Black/African American	1.5 (1)
White	92.4 (61)
Other	3.0 (2)
Ethnicity (*n* = 8 missing)	
Hispanic	14.3 (9)
Not Hispanic	85.7 (54)
DS type (*n* = 3 missing)	
Trisomy 21	91.2 (62)
Mosaicism	1.5 (1)
Translocation	2.9 (2)
Not sure	4.4 (3)
Premature birth (% yes; *n* = 5 missing)	21.2 (14)
Congenital heart defects (% yes; *n* = 3 missing)	75.0 (51)
Caregiver variable	% (*n*) or M (SD)
Primary caregiver age (mean/SD; *n* = 3 missing)	40.66 (6.04)
% primary caregiver education at least 1 year of college/tech training (*n*; *n* = 5 missing)	95.5 (63)
% annual household income (*n*; *n* = 3 missing)	
Below $50 000	10.3 (7)
$50 000–$100 000	26.5 (18)
Above $100 000	58.8 (40)
Did not wish to provide	4.4 (3)

DS, Down syndrome; SD, standard deviation.

**Table 2:** Descriptive analyses of executive function task performances

	Mean (SD)	Range (median)
Garage game (*n* = 62)		
Repetitive search rate	0.39 (0.39)	0–1.67 (0.29)
Snack delay (*n* = 71)		
Average time to dysregulated behaviour (s)	6.73 (5.88)	0.03–20.51 (5.44)
Total number of dysregulated behaviours	5.38 (1.89)	1–10 (5)
Adapted reverse categorisation		
Colour-congruent trials correct (pre-switch; *n* = 71)	7.28 (3.77)	0–10 (10)
Colour-incongruent trials correct (post-switch; *n* = 71)	4.00 (4.56)	0–10 (1)

SD, standard deviation.

**Table 3:** Descriptive analyses of adaptive behaviour at visits 1–3

	*n*	Mean (SD)	Range
Visit 1			
Adaptive Behaviour Composite SS	69	72.43 (6.85)	52–88
DLS SS	69	70.65 (9.63)	50–92
DLS: personal *v*-score	69	8.67 (2.42)	3–14
DLS: domestic *v*-score	62	11.31 (2.02)	8–16
DLS: community *v*-score	62	8.73 (2.11)	3–13
Visit 2			
Adaptive Behaviour Composite SS	47	71.13 (7.34)	49–88
DLS SS	47	67.87 (10.24)	50–96
DLS: personal *v*-score	47	8.26 (2.58)	3–14
DLS: domestic *v*-score	45	10.47 (2.07)	7–17
DLS: community *v*-score	45	8.44 (1.85)	5–12
Visit 3			
Adaptive Behaviour Composite SS	39	71.95 (8.46)	46–91
DLS SS	39	68.79 (11.70)	48–98
DLS: personal *v*-score	39	8.69 (3.50)	3–18
DLS: domestic *v*-score	39	10.64 (2.05)	7–16
DLS: community *v*-score	39	8.59 (2.14)	4–13

DLS, daily living skills; SD, standard deviation; SS, standard score.

**Table 4:** Nonparametric correlations between daily living skills subdomain scores and executive function performances

	Garage Game (*n*)	Snack Delay (*n*)	Adapted Reverse Categorisation (*n*)
Visit 1			
Personal *v*-score	−0.25* (61)	0.29* (69)	0.26* (69)
Domestic *v*-score	−0.24 (57)	0.39** (62)	0.25* (62)
Community *v*-score	−0.23 (57)	0.30* (62)	0.17 (62)
Visit 2			
Personal *v*-score	−0.38* (40)	0.50** (47)	0.24 (47)
Domestic *v*-score	−0.14 (40)	0.46** (45)	0.02 (45)
Community *v*-score	−0.29 (40)	0.49** (45)	0.26 (45)
Visit 3			
Personal *v*-score	−0.46** (35)	0.53** (39)	0.44* (39)
Domestic *v*-score	−0.38* (35)	0.34* (39)	0.33* (39)
Community *v*-score	−0.52** (35)	0.38* (39)	0.39* (39)

* *P* = 0.05.

** *P* = 0.01.

**Table 5:** Regression analyses for daily living skills standard scores at visits 1–3

	*B*	*β*	Sig. (p)
Daily living skills: visit 1 (*n* = 61)			
Garage game	3.58	0.15	0.326
Snack delay	0.64	0.40	0.003
Adapted reverse categorisation	0.59	0.28	0.047
Constant	62.15		<0.001
Daily living skills: visit 2 (*n* = 40)			
Garage game	1.33	0.05	0.750
Snack delay	0.93	0.56	<0.001
Adapted reverse categorisation	0.36	0.17	0.290
Constant	59.35		<0.001
Daily living skills: visit 3 (*n* = 35)			
Garage game	−4.14	−0.13	0.460
Snack delay	0.59	0.33	0.050
Adapted reverse categorisation	0.79	0.33	0.055
Constant	62.20		<0.001

Concurrent: *F*_3, 57_ = 5.02, *P* = 0.004, *R*^2^ = 0.21; 6-month follow-up: *F*_3, 36_ = 6.63, *P* = 0.001, *R*^2^ = 0.36; and 1-year follow-up: *F*_3, 31_ = 6.00, *P* = 0.002, *R*^2^ = 0.37.

## Data Availability

Data are available upon request from the corresponding author.
